# Induced pluripotent stem cell–derived mesenchymal stem cells enhance acellular nerve allografts to promote peripheral nerve regeneration by facilitating angiogenesis

**DOI:** 10.4103/NRR.NRR-D-22-00311

**Published:** 2024-09-06

**Authors:** Fan-Qi Meng, Chao-Chao Li, Wen-Jing Xu, Jun-Hao Deng, Yan-Jun Guan, Tie-Yuan Zhang, Bo-Yao Yang, Jian Zhang, Xiang-Ling Li, Feng Han, Zhi-Qi Ren, Shuai Xu, Yan Liang, Wen Jiang, Jiang Peng, Yu Wang, Hai-Ying Liu

**Affiliations:** 1 Department of Spine Surgery, Peking University People’s Hospital, Beijing, China; 2 Beijing Key Lab of Regenerative Medicine in Orthopedics, Institute of Orthopedics, Chinese PLA General Hospital, Beijing, China; 3 Co-Innovation Center of Neuroregeneration, Nantong University, Nantong, Jiangsu Province, China; 4 Department of Anesthesiology, Capital Medical University Xuanwu Hospital, Beijing, China; 5 Department of Orthopedics, The Fourth Medical Center of Chinese PLA General Hospital, Beijing, China; 6 Department of Orthopedics, First Affiliated Hospital, School of Medicine, Shihezi University, Shihezi, Xinjiang Uygur Autonomous Region, China; 7 National Clinical Research Center for Orthopedics, Beijing, China; 8 Department of Pain Treatment, People’s Hospital of Xinjiang Uygur Autonomous Region, Xinjiang Uygur Autonomous Region, China

**Keywords:** acellular nerve allograft, angiogenesis, bioluminescence imaging, conditioned medium, induced pluripotent stem cell–derived mesenchymal stem cells, micro-CT scanning, Microfil perfusion, peripheral nerve injury

## Abstract

Previous research has demonstrated the feasibility of repairing nerve defects through acellular allogeneic nerve grafting with bone marrow mesenchymal stem cells. However, adult tissue–derived mesenchymal stem cells encounter various obstacles, including limited tissue sources, invasive acquisition methods, cellular heterogeneity, purification challenges, cellular senescence, and diminished pluripotency and proliferation over successive passages. In this study, we used induced pluripotent stem cell-derived mesenchymal stem cells, known for their self-renewal capacity, multilineage differentiation potential, and immunomodulatory characteristics. We used induced pluripotent stem cell-derived mesenchymal stem cells in conjunction with acellular nerve allografts to address a 10 mm-long defect in a rat model of sciatic nerve injury. Our findings reveal that induced pluripotent stem cell-derived mesenchymal stem cells exhibit survival for up to 17 days in a rat model of peripheral nerve injury with acellular nerve allograft transplantation. Furthermore, the combination of acellular nerve allograft and induced pluripotent stem cell-derived mesenchymal stem cells significantly accelerates the regeneration of injured axons and improves behavioral function recovery in rats. Additionally, our *in vivo* and *in vitro* experiments indicate that induced pluripotent stem cell-derived mesenchymal stem cells play a pivotal role in promoting neovascularization. Collectively, our results suggest the potential of acellular nerve allografts with induced pluripotent stem cell-derived mesenchymal stem cells to augment nerve regeneration in rats, offering promising therapeutic strategies for clinical translation.

## Graphical abstract

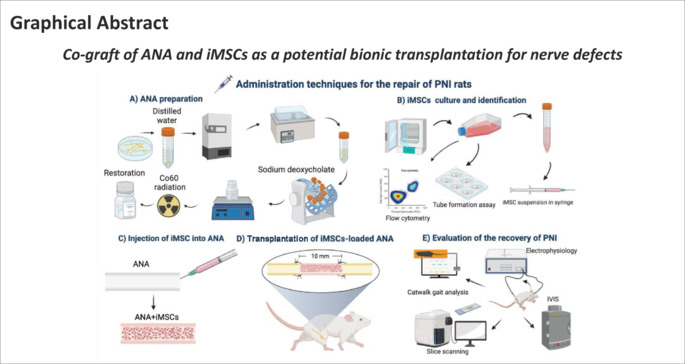

## Introduction

Peripheral nerve injury (PNI) refers to nerve injury or defect that leads to temporary or permanent sensory and/or locomotor impairment, which greatly affects patient quality of life (Kasper et al., 2020). The surgical treatment of PNI is a mainly direct end-to-end anastomosis, but this approach is limited to nerve defects without tension damage; otherwise, it causes local ischemia and substantially reduced regenerative effects (Kim et al., 2001; Schmidhammer et al., 2004). When the nerve defects cannot be surgically reconnected, it is important to build a bridge to cover the nerve gap for regeneration (Quan et al., 2021). Empty nerve conduits are useful for shorter gaps but are limited for longer gaps because of the lack of intraluminal biophysical guidance (Johnson et al., 2011). Autologous nerve transplantation is the golden standard for the treatment of PNI in clinical practice (Schmitte et al., 2010; Trehan et al., 2016). However, owing to the limited size and resources of autologous nerves, identifying novel approaches for nerve regeneration is urgent and critical (Schmidt and Leach, 2003). Moreover, the nerve removal process is invasive and may cause local inflammation and scarring (Kasper et al., 2020).

Acellular nerve allograft (ANA) has emerged as a preferable substitute for autologous nerves (Sun et al., 2010; Allgood et al., 2023). During the process of ANA preparation, cells, myelin, and disintegrating fragments are extracted by chemical detergent, and only neural bundle membrane and neural epineurium are retained. Thus, ANAs provide similar topographic, biochemical, and mechanical properties as endogenous nerves (Mohammad-Bagher et al., 2019). In our study, we demonstrated that ANAs are effective and safe for the repair of 1–5 cm nerve defects. Other reports showed that ANAs could accommodate critically-sized nerve defects (≥ 1 cm) in animal models and clinical applications (Jia et al., 2011; Moore et al., 2011; Li et al., 2016; Pan et al., 2019; Kasper et al., 2020).

Mesenchymal stem cells (MSCs) are adult stem cells that are mainly obtained from adult or fetal tissues or organs (Alizadeh et al., 2019). MSCs have several promising features for application purposes. However, there are still some obstacles to the use of adult organ- and tissue-derived MSCs, such as limited tissue sources, invasive harvesting methods, cellular heterogeneity, difficulty in purification, cellular senescence, and loss of pluripotency and proliferative capacity during serial passages (Liu et al., 2020; Elhussieny et al., 2021). Therefore, finding other high-quality MSCs to overcome the limitations of tissue-derived MSCs is critical.

Pluripotent stem cells have the potential to differentiate into a variety of cell tissues, including embryonic stem cells and induced pluripotent stem cells (iPSCs). iPSCs are usually derived from tissues such as blood mononuclear cells or skin and can be reprogrammed back to a pluripotent state, which allowing them to proliferate or differentiate into multiple cell types for cell therapy (Ikeda et al., 2014; Ohyama, 2019; Gong et al., 2021; Kamaraj et al., 2021; Onode et al., 2021; Voisin et al., 2023; Chen et al., 2024; Dong et al., 2024). The proliferative potential of induced MSCs (iMSCs) is much better than tissue-derived MSCs; iMSCs also maintain immune regulation and the application prospect of cell therapy (Ikeda et al., 2014; Yokoi et al., 2018). iMSCs can be obtained from a wide range of sources in a non-invasive manner and are easy to expand, which are advantages over matrix-derived MSCs. Importantly, iMSCs are less tumorigenic and less immunogenic compared with iPSCs (Ben-David and Benvenisty, 2011). iMSCs have been shown to be effective in the treatment of osteogenesis (Sheyn et al., 2016), chondrogenesis (Piñeiro-Ramil et al., 2019; Xu et al., 2020), angiogenesis (Hu et al., 2015), and inflammatory bowel disease (Yang et al., 2019). Therefore, iMSCs have emerged as a promising cell source for cell-based regenerative medicine (Mitsuzawa et al., 2020; Liang et al., 2021).

MSC-supplemented ANAs are a potential therapeutic method for segmental nerve defects (Bedar et al., 2022). ANAs and neural cells derived from adipose-derived MSCs hold great promise to replace autograft transplantation in peripheral nerve regeneration (Zhang et al., 2010). ANA may help MSCs remain at the nerve grafts to function. Wang et al. (2012) found that bone marrow stromal cell transplantation combined with chondroitin ABC enzyme promoted axonal regeneration and functional recovery after ANA repair of PNI rats. These findings may help to establish a new strategy for cell transplantation treatment of PNI.

In the present study, we investigated the effect of iMSCs and ANA co-graft on enhancing nerve regeneration in a PNI model. Our results provide evidence for the therapeutic effect of the co-graft on PNI and reveal pro-angiogenesis effects of iMSCs in nerve regeneration.

## Methods

### Cell culture

iMSCs were purchased from Nuwacell (Hefei, China; Cat# RC01005) and cultured in serum-free medium (ncMission hMSC Medium, Nuwacell, Cat# RP02010) at 37°C under a 5% CO_2_ humidified atmosphere. The medium was changed every 2–3 days. When the iMSCs reached 90% confluency, cells were harvested with 0.25% trypsin (Gibco, Grand Island, NY, USA) and plated at a density of 6 × 10^3^ cells/cm^2^. iMSCs in passages 3–4 were used in *in vivo* and *in vitro* experiments.

### Identification of induced pluripotent stem cell–derived mesenchymal stem cells

iMSC surface markers were determined by fluorescence-activated cell sorting analyses. iMSCs at passage 4 were harvested with 0.25% trypsin-ethylene diamine tetraacetic acid solution (Gibco). After washing with 0.01 M phosphate-buffered saline (PBS), the cells were labeled with fluorescein isothiocyanate anti-human CD90 (Thy1, BioLegend, San Diego, CA, USA, Cat# 328107, RRID: AB_893438), allophycocyanin (APC) anti-human CD73 (ecto-5′-nucleotidase, BioLegend, Cat# 344005, RRID: AB_1877158), PE/Cyanine7 anti-human leukocyte antigen DR (HLA-DR; BioLegend, Cat# 307615, RRID: AB_493589), phycoerythrin (PE) anti-human CD34 (BioLegend, Cat# 343605, RRID: AB_1732033), PerCP anti-human CD45 (BioLegend, Cat# 368505, RRID: AB_2566357), and brilliant violet 421 anti-CD105 (Endoglin; BioLegend, Cat# 800509, RRID: AB_2687062) following the manufacturer’s protocol and analyzed using the DxFLEX Flow Cytometer (Beckman Coulter, Brea, CA, USA). Data were analyzed using FlowJo (v.7.6.5; Becton, Dickinson & Company, Ashland, OR, USA).

### Enzyme-linked immunosorbent assay

iMSCs at passage 3 were grown in a high-glucose Dulbecco’s modified Eagle’s medium (Thermo Fisher, Waltham, MA, USA) supplemented with 10% fetal bovine serum and 1% penicillin/streptomycin. Once the cells reached 40%–50% confluency, the cells were shifted to 1% fetal bovine serum-high-glucose Dulbecco’s modified Eagle’s medium (as control medium). iMSC conditioned medium (iMSC-CM) was collected when iMSCs reached 90% confluence and centrifuged at 400 × *g* for 15 minutes to ensure complete removal of cellular debris. iMSC-CM was filtered through a 0.22 μm filter (Millipore, Boston, MA, USA). The concentrations of vascular endothelial growth factor (VEGF), nerve growth factor (NGF), and brain-derived neurotrophic factor (BDNF) were analyzed using enzyme-linked immunosorbent assay (ELISA) kits (Jiangsu Meimian Industrial Co., Ltd., Yancheng, China) following the manufacturer’s instructions.

### Tube formation *in vitro*

Human umbilical vein endothelial cells (2 × 10^4^ per well; Cat# iCell-h110, iCell Bioscience, Shanghai, China) were plated into the µ-Slide Angiogenesis, ibiTreat (Cat# 81506, ibidi, Gräfelfing, Germany) pre-coated with growth factor–reduced Matrigel (Cat# 354230, Corning, Corning, NY, USA) at 37°C for 1 hour and then incubated in iMSC-CM or control medium for 4 hours. Time serial images were obtained by a BZ-X800 microscope (Keyence, Osaka, Japan) for the observation of angiogenesis. The time window for observation was 4 hours. Tube length was calculated using ImageJ software (v-1.52q, National Institutes of Health, Bethesda, MD, USA) (Schneider et al., 2012).

### Preparation of acellular nerve allografts

All experimental procedures were performed following the institutional animal care guidelines and were approved by the Administration Committee of Experimental Animals of Chinese PLA General Hospital (Beijing, China; approval No. 2016-x09-07) on September 7, 2016. Adult female Sprague–Dawley rats (specific-pathogen-free level, 8 weeks old) were provided by SPF Biotechnology, Beijing, China (license No. SCXK 2019-0010). Male rats tended to fight other animals; therefore, to avoid unnecessary injury and death of animals, only female rats were used in this study.

**Figure 1:**
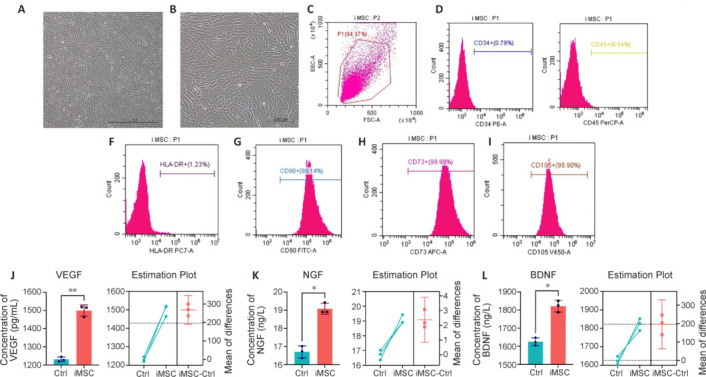
Morphology and characteristics of iMSCs. (A, B) Morphology of iMSCs at passage 4. The cells exhibited spindle-shaped, fibroblast-like morphology. Scale bars: 1 mm in A, 200 μm in B. (C–I) Flow cytometry analysis of iMSC surface antigens: CD34 (0.79%), CD45 (0.54%), HLA-DR (1.23%), CD90 (99.14%), CD73 (99.99%), and CD105 (99.90%). (J) VEGF concentration in iMSC-CM. (K) NGF concentration in iMSC-CM. (L) BDNF concentration in iMSC-CM. Data are presented as the mean ± SD. Enzyme-linked immunosorbent assays were performed in duplicate. *P < 0.05, **P < 0.01 (Student’s *t*-test). BDNF: Brain-derived neurotrophic factor; Ctrl: control; HLA-DR: human leukocyte antigen DR; iMSC-CM: conditioned medium of induced pluripotent stem cell-derived mesenchymal stem cells; iMSCs: induced pluripotent stem cell-derived mesenchymal stem cells; NGF: neurotrophic growth factor; VEGF: vascular endothelial growth factor.

Both sides of the sciatic nerve of 40 healthy rats (220–250 g) were harvested for decellularization. The nerve grafts were chemically and physically extracted into ANA, following previously described methods (Sondell et al., 1998). Nerve grafts were immersed in double distilled water and stored at –80°C for 12 hours, followed by thawing for 12 hours (**Figure 1A**). Nerve segments were decellularized with sodium deoxycholate (Sigma, St. Louis, MO, USA) at room temperature on a shaker at 60 r/min for 24 hours. Next, 0.01 M PBS was used to wash away remaining sodium deoxycholate. The procedures were performed under sterile conditions. The extracted ANAs were subjected to Co-60 radiation (Academy of Military Medical Sciences, Beijing, China) and preserved at –20°C (ANAs after radiation are shown in **Additional Figure 1A**).

### Construction of the bridge of sciatic nerve defects and transplantation of mesenchymal stem cells

Thirty adult female rats (200–220 g) were randomized into three groups (*n* = 10/group) (**Additional Table 1**). The animals were subjected to anesthesia with 1% pentobarbital sodium (intraperitoneal injection, 0.3 mL/100 g, Sigma). After sterilization of the skin, incision, and muscle splitting, the right sciatic nerve was exposed. A segment of the sciatic nerve was transected and a 10-mm-long gap was created. In the iMSC + ANA group, a 10-mm-long manicured ANA was injected with 20–30 μL of iMSC single-cell suspension (2 × 10^6^ cells/mL), and the nerve stumps were sutured with 10-0 silk (Ethicon, Somerville, MA, USA) (**Additional Figure 1B** and **C**). In the ANA group, a 10-mm-long manicured ANA was injected with only the medium of iMSCs and then transplanted into the sciatic nerve gap. In the Autograft group, a 10-mm-long segment of the sciatic nerve was transected and replanted via termino-terminal anastomosis into the nerve gap in the reverse direction. The postoperative rats were then housed in individually ventilated cages (five rats in each cage) with a 10/14-hour light/dark cycle. Food and water were offered *ad libitum*. All experiments were designed and reported following the Animal Research: Reporting of *In Vivo* Experiments (ARRIVE) guidelines (Percie du Sert et al., 2020).

**Additional Table 1:** Numbers of rats in experimental groups

Time points	Analysis	Groups		
		ANA	ANA+iMSC	Autograft
0-3 wk	BLI imaging of cells survival in PNI-ANA model		10	
12 wk	Catwalk analysis	8*	8*	8*
	Electrophysiology assessment	6*	6*	6*
	Wet weight of gastrocnemius muscle	6*	6*	6*
	Masson staining (muscle)	3^#^	3^#^	3^#^
	Microfil perfusion	3	3	3
	Immunofluorescence analysis of nerve graft	3^#^	3^#^	3^#^
	Toluidine blue staining of distal nerve			
		3	3	3

	Total	9	19	9

* means the number of rats not required to be executed until the last time point. ^#^means the samples of rats for multiple histological analyses, e.g., muscle tissue for Masson staining, nerve graft for immunofluorescence analysis, distal nerve stump for toluidine blue staining. ANA: Acellular nerve allograft; BLI: bioluminescence imaging; iMSCs: induced pluripotent stem cell-derived mesenchymal stem cells; PNI: peripheral nerve injury.

### Bioluminescence imaging

Bioluminescence imaging (BLI) was performed using the Spectrum *In Vivo* Imaging System Lumina LT Series III (PerkinElmer, Waltham, MA, USA) to explore the *in vivo* survival of iMSCs. A luciferase reporter gene-labeled iMSC cell line, capable of emitting light through bioluminescence, was utilized. Initially, a rat model with sciatic nerve defects was established, wherein the nerve stumps were sutured with ANAs. Subsequently, luciferase-labeled iMSCs were transplanted into the ANAs. To track the survival of iMSCs in the PNI-ANA models, we intraperitoneally injected the substrate luciferin, which reacts with luciferase in the presence of adenosine triphosphate (ATP) and oxygen to emit light. This bioluminescence occurs exclusively within live cells, and the intensity of light emission is linearly correlated with the number of labeled cells.

For *in vivo* tracking of iMSCs, luciferase-labeled iMSCs (Nuwacell) were injected into the rat model. We monitored the *in vivo* survival of luciferase-iMSCs every 1–2 days until no bioluminescence was detected in the rats, indicating the complete loss of iMSC viability. The rats were anesthetized with 2% isoflurane gas and received intraperitoneal injections of D-luciferin (150 mg/kg, dissolved in Dulbecco’s phosphate-buffered saline, PerkinElmer). Bioluminescence images were captured and photons data were quantitatively analyzed using *In Vivo* Imaging System Living Image software (PerkinElmer).

### Analysis of motor functional recovery

Motor functions were assessed using the CatWalk XT 10.6 (Noldus, Wageningen, Netherlands) every 4 weeks following surgery. The CatWalk XT system, known for its high sensitivity in evaluating animal gait and movement, automatically records footprints as rats move along a glass plate towards a target box. The system visualized the footprints and computes statistics regarding footprint size, time, and distance between tracks (Bozkurt et al., 2008). The sciatic functional index (SFI) were employed to gauge the rats’ motor function (Wang et al., 2018). It compromised hree measurements: (i) print length (PL), which represents the distance from the heel to the third toe; (ii) toe spread (TS), indicating the distance from the first to the fifth toe; and (iii) intermediate toe spread (ITS), reflecting the distance from the second to the fourth toe. All measurements were collected from the injured (E, for experimental) and non-injured (N, for normal) sides. SFI was calculated using the following equation: SFI = 109.5 (ETS – NTS)/NTS – 38.3 (EPL – NPL)/NPL + 13.3 (EIT – NIT)/NIT – 8.8 (Bain et al., 1989).

### Electrophysiological examination

Following 12 weeks of sciatic nerve recovery, all rats underwent an electrophysiological assessment to analyze the compound muscle action potential (CMAP) of the regenerated nerve, along with an evaluation of the conduction parameters of the operated graft nerve prior to sacrifice. Rats were anesthetized via intraperitoneal injection of pentobarbital sodium (intraperitoneal injection, 0.3 mL/100 g), and both sides of the sciatic nerve were exposed and isolated. The contralateral side served as the control. A ground electrode (Medlec Synergy; Oxford Instrument Inc., Oxford, UK) was subcutaneously positioned on the rats’ backs. Bipolar recording electrodes were inserted into the gastrocnemius muscle, while bipolar stimulating electrodes were placed at the proximal end of the nerve graft (**Additional Figure 1D**). A single electrical stimulus (intensity of 3.00 mA, duration of 0.1 ms, frequency of 10 Hz) was administered, and the latency period and wave amplitude were recorded.

### Statistical analysis

For animal group assignments, we determined experimental numbers based on prior expertise in the field to minimize the overall use of animals (Wang et al., 2021; Zhang et al., 2022). Specific animal numbers are detailed in **Additional Table 1**. After 12 weeks, all rats survived and data were assessed by two evaluators blinded to evaluation process. No fatalities occurred during the experiments. Data underwent analysis using GraphPad Prism 9.0 (GraphPad Software, San Diego, CA, USA) and were presented as the mean ± standard deviation (SD). Difference between two groups was assessed by Student’s *t*-test, while comparisons among multiple groups were conducted using one-way analysis of variance with Tukey’s *post hoc* test. A significant level of *P* < 0.05 was considered statistically significant.

## Results

### Morphology and characteristics of induced pluripotent stem cell–derived mesenchymal stem cells

iMSCs exhibited spindle-shaped and fibroblast-like morphology (**Figure 1A** and **B**).

Flow cytometry at passage 4 revealed positive expression of CD90 (99.14%), CD73 (99.99%), and CD105 (99.90%) and negative expression of CD34 (0.79%), CD45 (0.54%), and human leukocyte antigen-DR (HLA-DR; 1.23%) (**Figure 1C–I**). The low level of HLA-DR signifies low immunogenicity, in line with previous reports (Akle et al., 1981; Hu et al., 2015); Staining patterns for CD90, CD73, CD105, CD34, and CD45 were consistent with characteristics of MSCs (De Schauwer et al., 2012; Zahedi et al., 2017).

To assess the paracrine ability of iMSCs, we analyzed iMSC-CM, detecting the angiogenesis-related VEGF and neural growth factors NGF and BDNF (**Figure 1J–L**). HLA-DR, an MHC class II molecule (Robinson et al., 2015), exhibited low-level expression, confirming low immunogenicity (Wassmer and Berishvili, 2020). Other markers of iMSCs also exhibited typical MSCs characteristics (Numan et al., 2017; Chen et al., 2019).

### Conditioned medium of induced pluripotent stem cell–derived mesenchymal stem cell promotes angiogenesis *in vitro*

**Figure 2:**
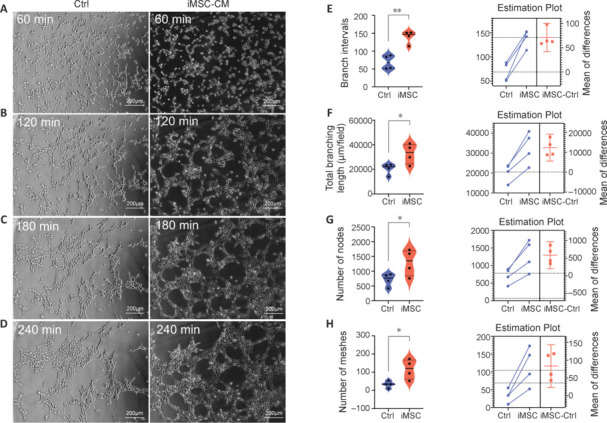
iMSC-CM promotes angiogenesis *in vitro*. (A–D) Images of HUVEC tube formation with different conditioned media (CM) at various time points. Scale bars: 200 μm. (E–H) Quantification of tube information assay (violin plots and estimation plots). (E) Number of branch intervals. (F) Total branching length. (G) Number of nodes. (H) Number of meshes. Tube formation assays were repeated four times. *P < 0.05, **P < 0.01 (Student’s *t*-test). Ctrl: Control (normal Dulbecco’s modified Eagle’s medium); HUVEC: human umbilical vein endothelial cell; iMSC-CM: conditioned medium of induced pluripotent stem cell-derived mesenchymal stem cell.

Tube formation assays were employed to investigate the angiogenic potential of iMSC-CM/every 4 hours, HUVECs migrated and formed tubular structures (**Figure 2A–D**). The iMSC-CM group displayed significantly higher branch intervals, total branching length, number of nodes, and number of meshes conpared with the control group (**Figure 2E–H**), indicating that iMSCs secreted pro-angiogenesis cytokines and promoted neovascularization.

### Survival of induced mesenchymal stem cells *in vivo*

**Figure 3:**
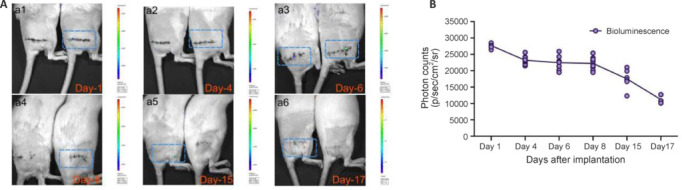
Survival time of iMSCs in the rat sciatic nerve injury model. (A) Bioluminescence signals at different time points following GFP-Luciferase-iMSC + ANA transplantation. The bioluminescence decreased immediately after cell transplantation and began to decline rapidly by the 8^th^ day post-transplantation. (B) Quantification of bioluminescence imaging. Data are expressed as mean ± SD (*n* = 3–10 rats/group). ANA: Acellular nerve allograft; GFP: green fluorescent protein; iMSC: induced pluripotent stem cell-derived mesenchymal stem cell; p: photons; sr: steradian.

Bioluminescence imaging (BLI) provided real-time monitoring of iMSCs survival *in vivo* (Land et al., 2014; Rathbun et al., 2017). Bioluminescence was observed in the surgical area on the first postoperative day, with distinct masses visible on the 4^th^, 7^th^, 10^th^, and 13^th^ postoperative days (**Figure 3A**). By the 17^th^ day post-surgery, only one rat exhibited weak bioluminescence. Quantification revealed a gradual decline, reaching the lowest value at 1.120e+04 p/sec/cm^2^/sr (**Figure 3B**). These data demontrated the survival of iMSCs in ANAs for 17 days after transplantation *in vivo*, highlighting their potential functional role in nerve regeneration.

### Co-graft of ANA and induced pluripotent stem cell–derived mesenchymal stem cells improves motor nerve recovery in rats with sciatic nerve injury

**Figure 4:**
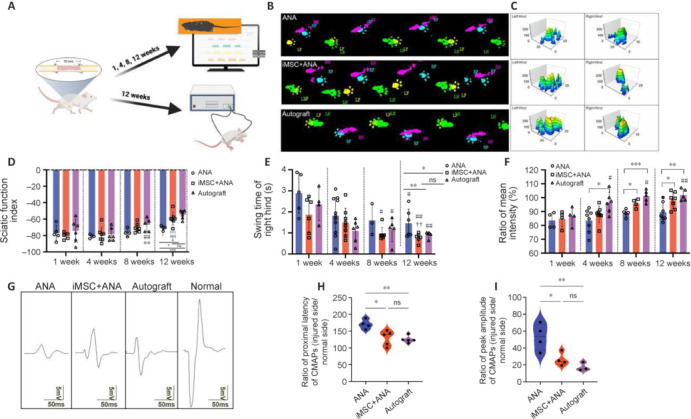
Effect of co-graft of ANA and iMSCs on motor nerve recovery in rats with sciatic nerve injury. (A) Schematic of the timeline for CatWalk gait and electrophysiological analysis of the rat model (created with Biorender.com). (B) Representative 2D footprints of each group. Red indicates the right hind (RH); green, the left hind (LH); blue, right front (RF); and yellow, the left front (LF). (C) Representative 3D paw pressure changes of the hind limbs in each group. X-axis represents the time when limbs hit the ground, and Y-axis represents the pressure of the limbs on the ground. (D) The trend of SFI changes over time. (E) Swing times of RH in each group. (F) Ratio of mean intensity of the 15 most intense pixels (experimental side/normal side). The statistical results of D-F are shown in Additional Table 2. (G) Representative waveforms of CMAP. (H) The ratio of latency of CMAP (injured side/normal side). (I) The ratio of peak amplitude of CMAP (injured side/normal side). Data are expressed as mean ± SD (*n* = 4–8 rats/group). *P < 0.05, **P < 0.01; #*P* < 0.05, ##*P* < 0.01, ###*P* < 0.001, *vs*. 1 week; †*P* < 0.05, ††*P* < 0.01, †††*P* < 0.001*,* *vs.* 4 weeks; &*P* < 0.05, *vs*. 8 weeks (one-way analysis of variance followed by Tukey’s *post hoc* test). ANA: Acellular nerve allograft; CMAP: compound muscle action potential; iMSCs: induced pluripotent stem cell-derived mesenchymal stem cells; ns: not significant.

Motor nerve recovery in rats with sciatic nerve injury was assessed through CatWalk gait analyses conducted at 4-week intervals until the study endpoint of 12 weeks (**Figure 4A**). To enhance the characteristics of paw prints and foot patterns, both two-dimensional (2D) and three-dimensional (3D) visualizations were employed (**Figure 4B** and **C**). The right hind RH paw print in the ANA group appeared slimmer and less distinct compared with the iMSC + ANA group (**Figure 4B**). In the 3D footprint map, the iMSC + ANA group exhibited larger footprints than the ANA group, although the Autograft group displayed the highest intensity (**Figure 4C**). During the initial 4 weeks post-surgery, all groups experienced a declined in Sciatic Functinal Index, followed by subsequent increase after 4 weeks (**Figure 4D**). At the final observation point, the SFI in the iMSC + ANA group demonstrated a superior recovery compared to the ANA group. The swing times of RH in all groups showed a decreasing trend, signifying increased movement speed, with the iMSC + ANA group showing a statistically significant difference from the ANA group at the endpoint (**Figure 4E**). The ratio of the mean intensity of the 15 most intense pixels of hindpaws (experimental side/normal side) displayed an increasing trend, and the iMSC + ANA group exhibited a statistically significant difference from the ANA group, indicating better intensity in the right hind of the iMSC + ANA group compared with the ANA group (**Figure 4F**).

**Additional Table 2:** Statistical results of SFI changes, swing times of right hind and ratio of mean intensity

Group	Statistical results in different time points	Statistical results in different groups
**Figure 4D**		
ANA group	12 wk vs. 4 wk:P = 0.0305	12 weeks:
iMSC+ANA group	12 wk vs. 1 wk: P = 0.0003	iMSC+ANA vs. ANA: P = 0.026
	12 wk vs. 4 wk: P = 0.0002	Autograft vs. ANA: P = 0.0032
	12 wk vs. 8 wk: P = 0.0320	iMSC+ANA vs. Autograft: P = 0.3839
Autograft group	8 wk vs. 4 wk: P = 0.0094	
	8 wk vs. 1 wk: P = 0.0017	
**Figure 4E**		
ANA group	12 wk vs. 1 wk: P = 0.0480	12 weeks:
iMSC+ANA group	8 wk vs. 1 wk: P = 0.0452	iMSC+ANA vs. ANA: P = 0.0071
	12 wk vs. 1 wk: P = 0.0058	Autograft vs. ANA: P = 0.0162
	12 wk vs. 4 wk: P = 0.0120	iMSC+ANA vs. Autograft: P = 0.7318
Autograft group	8 wk vs. 1 wk: P = 0.0093	
	8 wk vs. 1 wk: P = 0.0063	
**Figure 4F**		
ANA group	ns	4 wk:
iMSC+ANA group	ns	ANA vs. Autograft: P = 0.0102
Autograft group	4 wk vs. 1 wk: P = 0.0112	8 wk:
	8 wk vs. 1 wk: P = 0.0209	ANA vs. iMSC+ANA: P = 0.0335
	12 wk vs. 1 wk: P = 0.0052	ANA vs. Autograft: P = 0.0005
		12 wk:
		ANA vs. iMSC+ANA: P = 0.0497
		ANA vs. Autograft: P = 0.0076

After 12 weeks post-surgery, electrophysiological evaluation was conducted, recording the peak amplitude and latency of CMAPs were recorded. The iMSC + ANA group exhibited a markedly higher CMAP amplitude than the ANA group and was comparable to the Autograft group (**Figure 4G**). Both iMSC + ANA and Autograft groups showed waves more akin to the control than the ANA group. As depicted in **Figure 4H**, the ratios of latency of CMAPs in the iMSC + ANA and Autograft groups were significantly lower compared with the ANA group (*P* < 0.01). The ratio of CMAP amplitude in the iMSC + ANA group was significantly higher than the ANA group, with the Autograft group reaching (53.03 ± 15.76)% (**Figure 4I**). The iMSC + ANA group exhibited markedly decreased CMAPs compared with the contralateral nerve. in comparison with the ANA group, both the iMSC + ANA and Autograft groups displayed superior conduction of CMAPs.

### Co-graft of acellular nerve allograft and induced pluripotent stem cell–derived mesenchymal stem cells improves atrophy of the gastrocnemius in rats with sciatic nerve injury

**Figure 5:**
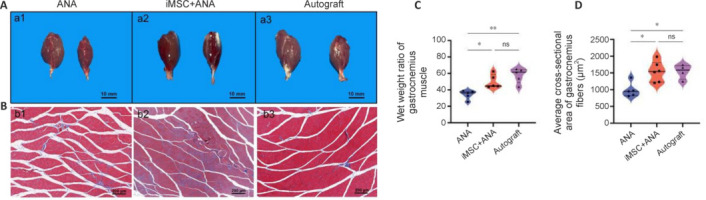
Effect of co-graft of ANA and iMSCs on the target muscles of the regenerated nerves in rats with sciatic nerve injury after 12 weeks. (A) General view of the gastrocnemius muscle in each group. The left side is the healthy side, and the right side is the operated side. (B) Representative image of Masson trichromatic staining of rat gastrocnemius. Scale bars: 10 mm in A, and 200 μm in B. (C) The wet weight ratio of the gastrocnemius muscle (injured side/normal side) in each group. The raw data is shown in Additional Table 3. (D) The mean cross-sectional area of the gastrocnemius muscle fiber. Data are expressed as mean ± SD (*n* = 5–6 rats/group). *P < 0.05, **P < 0.01 (one-way analysis of variance followed by Tukey’s *post hoc* test). ANA: Acellular nerve allograft; iMSCs: induced pluripotent stem cell-derived mesenchymal stem cells; ns: not significant.

A macroscopic view of the gastrocnemius muscle 12 weeks post-surgery is presented in **Figure 5A**. All rats retained their right hind limb without any amputations. In the ANA group, two out of six rats exhibited severe ulcers on the right hind limb, while in the Autograft and iMSC + ANA groups, only one out of six rats displayed a severe ulcer. Varied degrees of muscle atrophy were noted among the groups. At 12 weeks, muscle atrophy in the iMSC + ANA and Autograft groups was less pronounced compared with the ANA group (**Figure 5A**). Cross-sectional analysis of muscle in the iMSC + ANA and Autograft groups revealed a larger fiber area and reduced connective tissue (**Figure 5B**). The wet weight ratio of the iMSC + ANA and Autograft groups was significantly higher than that of the ANA group (**Figure 5C**). Overtime, an increase in gastrocnemius muscle fibers was observed. At 12 weeks post-surgery, the average cross-sectional area of gastrocnemius fibers in the iMSC + ANA group was significantly higher than that in the ANA group, comparable to the Autograft group (*P* < 0.05; **Figure 5D**).

**Additional Table 3:** Weight of gastrocnemius muscles

	Left (g)	Right (g)	Percentage (right/left)
ANA			
Sample 1	1.85	0.67	36.22
Sample 2	1.72	0.65	37.79
Sample 3	1.71	0.56	32.75
Sample 4	1.54	0.39	25.32
Sample 5	1.78	0.69	38.76
iMSC + ANA			
Sample 1	1.96	0.88	44.90
Sample 2	1.71	0.94	54.97
Sample 3	1.87	0.82	43.85
Sample 4	1.65	1.03	62.42
Autograft			
Sample 1	1.6	0.7	43.75
Sample 2	1.99	1.3	65.33
Sample 3	1.92	1.23	64.06
Sample 4	1.63	0.88	53.99
Sample 5	1.79	1.1	61.45

ANA: Acellular nerve allograft; iMSCs: induced pluripotent stem cell-derived mesenchymal stem cells.

### Co-graft of acellular nerve allograft and induced pluripotent stem cell–derived mesenchymal stem cells enhances the vascularization and regeneration of sciatic nerve injury

**Figure 6:**
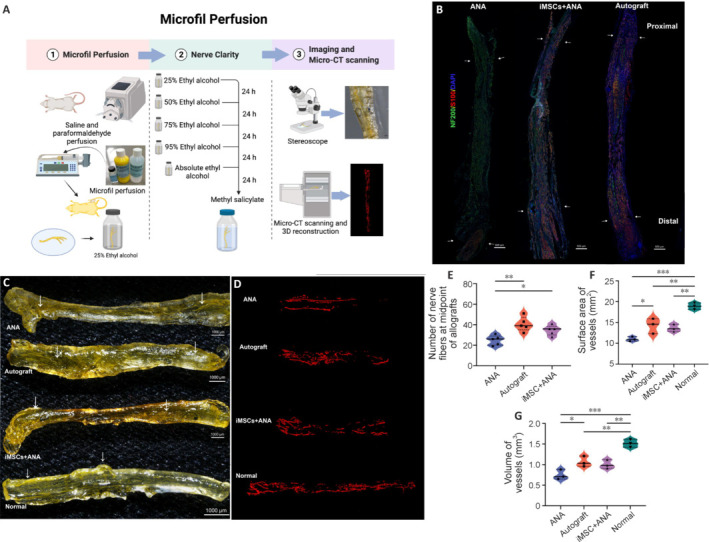
Effect of co-graft of ANA and iMSCs on the vascularization around regenerated nerves in rats with sciatic nerve injury after 12 weeks. (A) Illustration of Microfil methods. (B) Morphological images of Microfil-perfused nerves. White arrow: Microfil-perfused microvessel. (C) 3D reconstruction image of Microfil-perfused nerves. (D) Immunofluorescence staining of the regenerated tissues. NF200 (a marker for neurofilament, green), S100 (a marker for SCs, red), and nuclei (DAPI, blue). White arrow: suture knots. Scale bars: 500 μm in B, 1000 μm in C, and 500 μm in D. (E) Number of nerve fibers at the midpoint of allografts. (F) Surface area of vessels of Microfil-perfused nerves. (G) Volume of vessels of Microfil-perfused nerves. Data are expressed as mean ± SD (*n* = 5–6 rats/group). *P < 0.05, **P < 0.01, ***P < 0.001 (one-way analysis of variance followed by Tukey’s *post hoc* test). 3D: Three-dimensional; ANA: acellular nerve allograft; CT: computed tomography; DAPI: 4′,6-diamidino-2-phenylindol; iMSCs: induced pluripotent stem cell-derived mesenchymal stem cells; NF200: neurofilament protein 200.

The nerve grafts underwent Microfil perfusion and tissue cleaning (**Figure 6A**). Macroscopic images illustrated successful replacement of the blood with Microfil, revealing clear yellow vessels under a stereoscope (**Figure 6B**). Subsequent to CT scanning, images underwent reconstruction and rendering with Analyze software. Improved vascularization was evident in the normal nerve, iMSC + ANA, and Autograft groups compared with the other groups (**Figure 6C**). Immunofluorescence staining of the longitudinal section of nerve grafts is presented in **Figure 6D**. A statistically significant difference in the number of neurofilaments in the middle of the nerve grafts was observed between the ANA and iMSC + ANA groups (*P* = 0.0444), with an extremely significant difference between the Autograft and ANA groups (*P* = 0.0023; **Figure 6E**). For the statistical analysis of neovascularization, the surface area and volume of vessels were calculated. The largest average surface area of vessels was found in the Autograft group, followed by the iMSC + ANA and ANA groups (**Figure 6F**). Similarly, the largest volume of vessels was found in the Autograft group, followed by the iMSC + ANA and ANA groups (**Figure 6G**). While no statistically significant difference exhisted between iMSC + ANA and ANA groups (*P* = 0.0914), statistical differences were evident between the ANA and Autograft groups in both area and volume parameters (*P* = 0.0057).

### Co-graft of acellular nerve allograft and induced pluripotent stem cell–derived mesenchymal stem cells improves myelin sheath regeneration in rats with sciatic nerve injury

**Figure 7:**
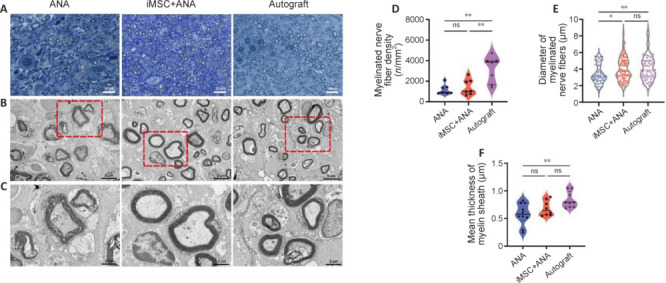
Effect of co-graft of ANA and iMSCs on regenerated myelin in rats with sciatic nerve injury after 12 weeks. (A) Toluidine blue staining of the distal nerve graft. (B) Transmission electron microscope images of myelin sheath in the distal nerve graft. (C) Enlarged transmission electron microscopy images of red boxes in B. Scale bars: 20 μm in A, 5 μm in B and 2 μm in C. (D) The average number per unit area of the myelin sheath. (E) The average diameter of the myelin sheath. (F) The average thickness of the myelin sheath. Data are expressed as mean ± SD (*n* = 3 rats/group). *P < 0.05, **P < 0.01 (one-way analysis of variance followed by Tukey’s *post hoc* test). ANA: Acellular nerve allograft; iMSCs: induced pluripotent stem cell-derived mesenchymal stem cells; ns: not significant.

Toluidine blue staining of transverse semi-thin sections of the distal nerve stumps in different groups revealed varying degrees of axonal regeneration. In the iMSC + ANA and Autograft groups, myelinated fibers appeared densely packed and evenly arranged, surrounded by a well-formed myelin sheath. Both groups exhibited similar numbers and roughly equivalent diameters of myelinated nerve fibers (**Figure 7A**). Electron microscopy further illustrated that the ANA group displayed sparse myelin fiber layers with a loose arrangement, while the iMSC + ANA and Autograft groups exhibited dense myelination (**Figure 7B** and **C**). The density of regenerated myelinated nerve fibers, the average diameter of the myelin sheath, and the average thickness of the myelin sheath followed the order of Autograft, iMSC + ANA, and ANA groups (**Figure 7D–F**). A significant difference in myelin sheath thickness was noted between the Autograft and ANA groups (*P* = 0.0072), while no significance was observed between the iMSC + ANA and ANA groups (**Figure 7D**). The diameter of myelinated nerve fibers in the iMSC + ANA group (3.947 ± 1.379) was significantly smaller than that in the Autograft (*P* = 0.7426) and ANA groups (**Figure 7E**). No statistically significant difference in the density of myelinated nerve fibers between the iMSC + ANA and ANA groups (*P* = 0.9455; **Figure 7F**). Immunofluorescence staining and electron microscopy results collectively demonstrated that iMSCs and ANA collaboratively enhanced neurofilament development and myelination in nerve regeneration.

## Discussion

MSCs are notable for secretion of cytokines, crucial in tissue regeneration and organ repair. In contrast to other MSCs sources with limited origins, invasive harvesting methods, and constrained proliferation and differentiation capabilities, iMSCs present numerous advantages (Sheyn et al., 2016). These cells can be derived from diverse sources, exhibiting low immunogenicity and promising regenerative potential (Kwon et al., 2020). Alternative to autografts, such as artificial nerve conduits (ANAs), offer longitudinal guidance channels, vital for Schwann cell migration and the formation of Büngner bands (Pan et al., 2020). Previous studies underscore ANAs’ efficay in repairing nerve lesions when combined with MSCs (Li et al., 2016; Pan et al., 2019). Our investigation of the therapeutic impact of iMSCs and ANA in nerve recovery involved a co-graft of these in a rat sciatic nerve defect model, revealing a substantial improvement in nerve regeneration.

Typical MSC transplantation invloves the systematic administration of a large number of MSCs, facilitating widespread distribution. These cells rapidly migrate to damaged organs releasing stress-induced therapeutic molecules before being cleared by the body, functioning through a “hit and run” mechanism (von Bahr et al., 2012). Despite the therapeutic benefits suggested by previous studies, the inability to accurately locate MSC secretome and predict its fate poses challenges in determining target cell types and therapeutic effects.

In stem cell therapy, BLI is recognized for providing insights into cell survival, migration, and immunogenicity in living animals (de Almeida et al., 2011). Our BLI data indicated that iMSCs survived for approximately 20 days in the extracellular matrix. Comparatively, a prior study demonstrated a 29-day lifespan for adipose-derived MSCs in ANA transplantation (Rbia et al., 2019). Unpublished data from our study with human umbilical cord–derived MSCs in peripherally inserted ANAs (PNI-ANA) rat models revealed an average life of 21 days. Recent research (Bae et al., 2022), corroborated this survival lifespan for injected MSCs. The authors of that study compared MSC attachment efficiency in acellular nerve allografts for peripheral nerve regeneration. Notably, even with the most efficient attachment method in ANAs (Matrigel), MSC nuclei were detectable after one week and dwindled after two weeks, with minimal traces after four weeks. This underscores the dependence of cell-based therapy efficiency on an adequate cell quantity and efficient delivery to damaged tissue, emphasizing the need to explore methods for obtaining sufficient iMSCs in ANAs *in vivo*.

Prior studies have demonstrated that MSC-CM mirrors the therapeutic effects of MSCs, supporting a “paracrine hypothesis” and the “hit-and-run” mechanism (Gnecchi et al., 2008; von Bahr et al., 2012). Liang et al. (2021) reported that iMSC-CM promoted cutaneous wound healing through the secretion of growth factors. Furthermore, iMSC-CM demonstrated superiority to conditioned medium of MSCs derived from umbilical cord MSCs in accelerating wound closure and enhancing angiogenesis via the ERK pathway (Yokoi et al., 2021).

Our ELISA results revealed NGF, BDNF, and VEGF in iMSC-CM. Neurotrophic factors such as BDNF and NGF accelerate and trigger axonal regrowth, which contributes to accelerated nerve repair (Ranganath et al., 2012). The concentration of VEGF in iMSC-CM was higher than that of the control group by approximately 200 pg/mL. VEGF is a critical regulator in angiogenesis, SC invasion, and myelin sheath regeneration (Hobson et al., 2000). Angiogenesis is essential for tissue repair and requires an adequate network of blood vessels to supply the injured tissue with blood and growth factors (Caplan and Correa, 2011; Maacha et al., 2020). A previous study highlighted macrophage-derived VEGF-ɑ induced angiogenesis and preceded the migration of SCs in the nerve bridge (Cattin et al., 2015). The underscoring the role of neovascularization in guiding SCs during nerve regeneration.

Behavior tests and electrophysiological assessments were conducted to evaluate motor function recovery. The motor recovery parameters of the iMSC + ANA group were comparable to those of the Autograft group. The 2D and 3D limb prints could intuitively show the size of the footprints and the intensity of the paw prints. The results of swing time of RH and the ratio of mean intensity of the 15 most intense pixels of hinds in the iMSC + ANA group were relatively encouraging results. While SFI is the most commonly used index for motor recovery of the sciatic nerve, the results were not satisfying. The SFI of the 4^th^ week was lower than that of the 1^st^ week; after the 4^th^ week, the SFI started to rise. We assume this may be because of Wallerian degeneration, which includes axon necrosis, myelin decomposition and disappearance, nerve sheath hyperplasia, and a series of metamorphosis events in the distal segment of the injured nerve (Zvara et al., 2010; Jung et al., 2011). Wallerian degeneration is usually a long-term process following nerve transection that happens in 1 or 2 days after nerve injury and lasts for 4 weeks (Conforti et al., 2014). At the 4^th^ week, the regenerated nerve fibers do not innervate into the targeted muscles, which would cause gradual muscle atrophy and reduced systolic function. Therefore, we assume the 4^th^ week after nerve injury is a turning point for SFI to rise.

From the perspective of nerve fiber regeneration, the Autograft group exhibited certain advantages in terms of continuity of regenerated axons, morphological arrangement of myelinated nerve fibers, diameter of myelinated nerve fibers, and thickness of the myelin sheath. The results of the iMSC + ANA group were comparable to those of the Autograft group regarding axon continuity, diameter of regenerated nerve fiber, and thickness of myelin sheath. Although nerve grafts in all groups restored the continuity of sciatic nerve defects, there were differences in the quality of regeneration. The first difference was the morphology, distribution, and diameter of regenerated myelinated nerve fibers, as wel as the distribution of fibrous connective tissue and unmyelinated nerve fibers among the groups. The ANA group displayed irregular, smaller myelinated nerve fibers filled with fibrous connective tissue compared to the Autograft group. In contrast, the iMSC + ANA group showed regenerated myelinated nerve fibers with regular morphology, more uniform distribution, and a diameter closer to that of the Autograft group, though some fibrous connective tissue and unmyelinated nerve fibers remained, affecting the efficiency of electrical signal transduction. The second difference was in the thickness of the myelin sheath of regenerated myelinated nerve fibers. The density of myelinated nerve fibers in the iMSC + ANA and ANA groups was significantly lower than that in the Autograft group. This may be due to the requirement for the restoration of myelin sheath thickness and density to form Ranvier’s nodes, which are critical for the normal function of the nerve fibers (Ikeda et al., 2000).

iMSCs promote angiogenesis through their paracrine actions. In a mouse model of hindlimb ischemia, iMSC transplantation into ischemic limbs improved perfusion recovery and limb neovascularization (Lian et al., 2010). MSCs can promote neovascularization and perfusion by secreting paracrine factors and functioning as perivascular precursor cells (Choi et al., 2013).

We demonstrated the angiogenesis capability of the iMSC and ANA co-graft. Tube formation assay revealed that iMSC-CM improved vascularization *in vitro*. Microfil perfusion was used to construct a three-dimensional reconstructive vessel model, providing a macroscopic visualization of angiogenesis in regenerated nerves. Although there was no significant difference between the iMSC + ANA and ANA groups, the average surface area and volume of vessels in the iMSC + ANA group were higher than those in the ANA group. Microfil only perfuses vessels with a diameter larger than 10 μm (Wang et al., 2016; Juhl et al., 2019).

Zhu et al. (2017) found that the growth of microvessels starts at both stumps of nerve. Similarly, in our study, vascularization at the ends of transected nerve was denser than that in the middle of grafts. Besides the angiogenic regulation of iMSCs, ANA might also provide a pro-angiogenesis environment for nerve regeneration (Muheremu et al., 2016). As a nerve extracellular matrix, ANA plays an important role in regulating angiogenesis and homeostasis (Marchand et al., 2019). ANA provides a hypoxic environment for nerve regeneration and stimulates the secretion of pro-angiogenesis factors, such as VEGF (Gao et al., 2013; Saffari et al., 2021). The basal layer of ANA is also crucial for Schwann cell migration and axon regeneration. One study used enzyme digestion to decellularize peripheral nerve and reduced the detrimental effects of ionic detergent on the ECM composition (Chato-Astrain et al., 2020b).

The specific role of iMSCs in the ANA after transplantation remains to be fully elucidated. From previous studies and our research, we speculate that iMSCs may migrate into the micro-channels of ANA and replace the injured or dead cells. The chemotaxis effect and attractive forces exerted by various chemokines released after Wallerian degeneration and tissue degeneration drive iMSCs to concentrate at the damage site. These accumulated iMSCs may secrete a large number of nutrients and pro-angiogenesis cytokines to promote axon regeneration and vascularization (Rani et al., 2015).

Our results provide proof of concept that the combination of iMSCs and ANA has the potential to promote nerve regeneration, paticularly in relation to angiogenesis. However, the mechanisms of iMSCs on axon regeneration and angiogenesis have not been thoroughly elucidated. Transcriptome and proteomic analyses are required to identify the specific roles of iMSCs in neural regenerative process and angiogenesis, which will be the focus of our next study. Due to limited proliferative effects and aggregation ability, iMSCs can only survive in ANA for approximately 20 days. To provide sufficient cells, secondary or multiple injections in cell therapy may be necessary, which could provide more cells for the neural regenerative process. Assessing the feasibility of multi-injection therapy is beneficial for translating cell therapy from “bench-to-bed”.

In conclusion, we demonstrated the suitability of iMSCs as a promising alternative stem cell source for future PNI therapies. We found that iMSCs survived for 17 days in ANA tissue *in vivo*. VEGF, NGF, and BDNF secreted by iMSCs might play an important role in angiogenesis and proliferation of Schwann cells in the microenvironment of nerve regeneration. Importantly, the co-graft of ANA and iMSCs is promising for bionic transplantation for nerve defects and represent a novel therapeutic application. However, we lack the mechanistic studies on interaction between iMSCs and ANAs *in vitro*. Future studies involving multiple time points, longer-term follow-up, and transcriptome and proteomic analyses should explore the specific mechanisms in nerve regeneration.

## Additional files:

***Additional Figure 1:*** *Establishment of ANA-iMSC co-graft and peripheral nerve injury repair model.*

Additional Figure 1 Establishment of ANA-iMSC co-graft and peripheral nerve injury repair model.
(A) Sterilized ANA (Co-60 radiation). (B) Construction of sciatic nerve injury model with ANA-iMSCs co-grafts. (C) ANA-iMSCs co-grafts (under microscope, intraoperative). (D) Electrophysiological analysis of regenerated nerve. ANA: Acellular nerve allograft; iMSCs: induced pluripotent stem cell-derived mesenchymal stem cells.

***Additional Table 1:*** *Numbers of rats in experimental groups.*

***Additional Table 2:*** *Statistical results of SFI changes, swing times of right hind and ratio of mean intensity.*

***Additional Table 3:*** *Weight of gastrocnemius muscles.*

## Data Availability

*All relevant data are within the paper and its Additional files*.
