# Supplementary figures and images for: Induced pluripotent stem cell–derived mesenchymal stem cells enhance acellular nerve allografts to promote peripheral nerve regeneration by facilitating angiogenesis
Source: Neural Regen Res. 2024 Sep 6;21(5):2050–9. doi: 10.4103/NRR.NRR-D-22-00311 (PMC12694729; doi:10.4103/NRR.NRR-D-22-00311)

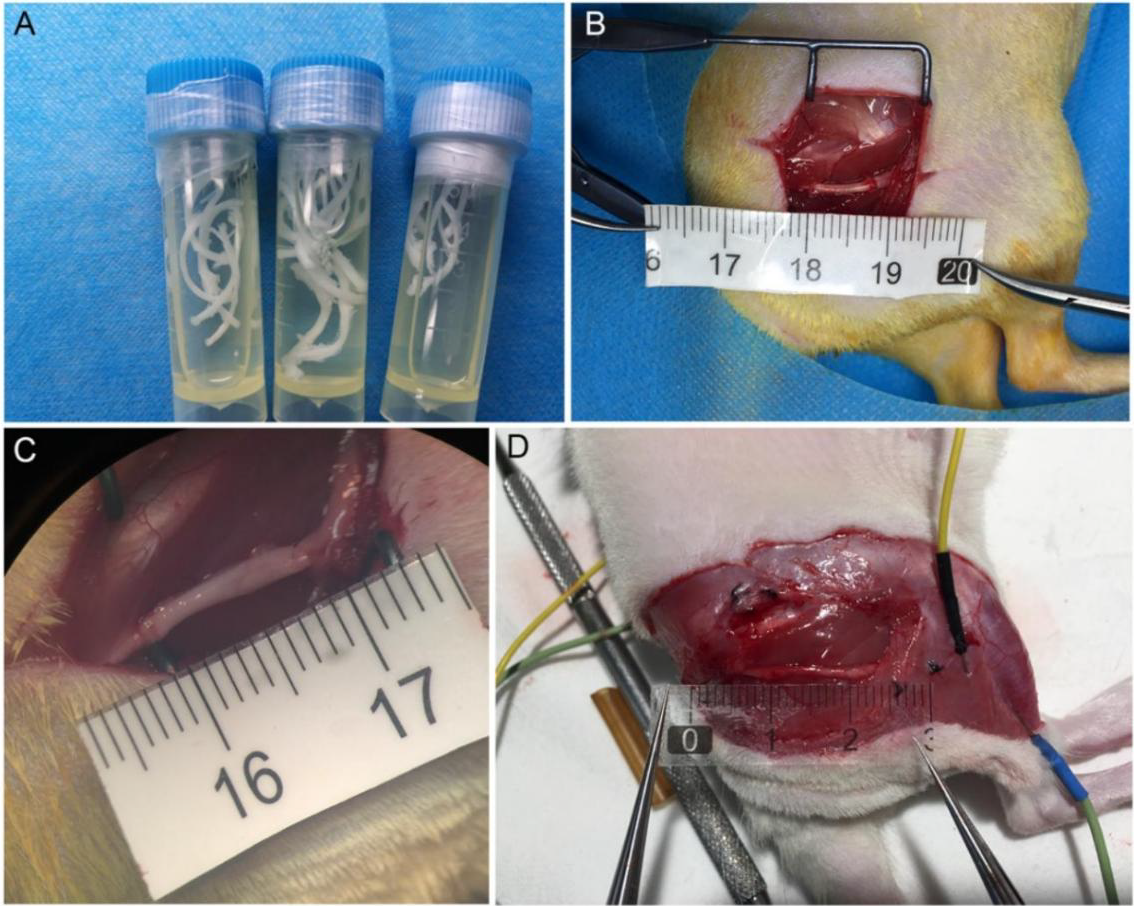

Supplement: Supplementary file 1 [file NRR-21-2050_Suppl1.tif]
